# Chromatic pupillometry for evaluating melanopsin retinal ganglion cell function in Alzheimer’s disease and other neurodegenerative disorders: a review

**DOI:** 10.3389/fpsyg.2023.1295129

**Published:** 2024-01-08

**Authors:** Martina Romagnoli, Giulia Amore, Pietro Avanzini, Valerio Carelli, Chiara La Morgia

**Affiliations:** ^1^IRCCS Istituto delle Scienze Neurologiche di Bologna, Programma di Neurogenetica, Bologna, Italy; ^2^Dipartimento di Scienze Biomediche e Neuromotorie, Università di Bologna, Bologna, Italy; ^3^CNR, Istituto di Neuroscienze, Parma, Italy; ^4^IRCCS Istituto delle Scienze Neurologiche di Bologna, UOC Clinica Neurologica, Bologna, Italy

**Keywords:** chromatic pupillometry, melanopsin retinal ganglion cells, Alzheimer’s disease, pupil, post-illumination pupil response, neurodegeneration

## Abstract

The evaluation of pupillary light reflex (PLR) by chromatic pupillometry may provide a unique insight into specific photoreceptor functions. Chromatic pupillometry refers to evaluating PLR to different wavelengths and intensities of light in order to differentiate outer/inner retinal photoreceptor contributions to the PLR. Different protocols have been tested and are now established to assess *in-vivo* PLR contribution mediated by melanopsin retinal ganglion cells (mRGCs). These intrinsically photosensitive photoreceptors modulate the non-image-forming functions of the eye, which are mainly the circadian photoentrainment and PLR, via projections to the hypothalamic suprachiasmatic and olivary pretectal nucleus, respectively. In this context, chromatic pupillometry has been used as an alternative and non-invasive tool to evaluate the mRGC system in several clinical settings, including hereditary optic neuropathies, glaucoma, and neurodegenerative disorders such as Parkinson’s disease (PD), idiopathic/isolated rapid eye movement sleep behavior disorder (iRBD), and Alzheimer’s disease (AD). The purpose of this article is to review the key steps of chromatic pupillometry protocols for studying *in-vivo* mRGC-system functionality and provide the main findings of this technique in the research setting on neurodegeneration. mRGC-dependent pupillary responses are short-wavelength sensitive, have a higher threshold of activation, and are much slower and sustained compared with rod- and cone-mediated responses, driving the tonic component of the PLR during exposure to high-irradiance and continuous light stimulus. Thus, mRGCs contribute mainly to the tonic component of the post-illumination pupil response (PIPR) to bright blue light flash that persists after light stimulation is switched off. Given the role of mRGCs in circadian photoentrainment, the use of chromatic pupillometry to perform a functional evaluation of mRGcs may be proposed as an early biomarker of mRGC-dysfunction in neurodegenerative disorders characterized by circadian and/or sleep dysfunction such as AD, PD, and its prodromal phase iRBD. The evaluation by chromatic pupillometry of mRGC-system functionality may lay the groundwork for a new, easily accessible biomarker that can be exploited also as the starting point for future longitudinal cohort studies aimed at stratifying the risk of conversion in these disorders.

## 1 Introduction

Over the last decades, the pupil has been considered a window into brain functions for a wide range of clinical settings, including neurodegenerative disorders (Hall and Chilcott, 2018). More specifically, the evaluation of pupil size and dynamics to light stimulation, i.e., pupillary light reflex (PLR), by chromatic pupillometry may provide a unique insight into specific photoreceptor functions (Park et al., 2011; Hall and Chilcott, 2018).

The pupil has a large dynamic range and is controlled by the antagonistic actions of the iris sphincter and dilator muscles, which are innervated by the parasympathetic and sympathetic nervous systems, respectively (Hall and Chilcott, 2018). Average pupil diameter is influenced by factors such as age, sex, iris color, retinal and/or optic nerve health, and optical media clarity, but the most powerful determinant of pupil size is the ambient light level (Hall and Chilcott, 2018).

The term PLR refers to a reflex that controls the constriction and subsequent dilation of the pupil in response to changes in light intensity, and its dynamics follow a pattern composed of four phases (response latency, maximum constriction, pupil escape, and recovery) that can be influenced by the duration, intensity and spectral composition of the light (Hall and Chilcott, 2018). In addition to these PLR dynamic phases, during light stimulation, which is mainly driven by rods and cones, it can be also recognized a slow and sustained component (Park et al., 2011; Adhikari et al., 2016; Joyce et al., 2016) named post-illumination pupil response (PIPR) (Berson et al., 2002; Park et al., 2011). The PIPR is mainly dependent on melanopsin retinal ganglion cells (mRGCs) (Kardon et al., 2009; Park et al., 2011; Adhikari et al., 2015), a class of retinal ganglion cells (RGCs) expressing the photopigment melanopsin, which are intrinsically photosensitive and project to the olivary pretectal nucleus (OPN), the pupillomotor center (Berson et al., 2002; Hattar et al., 2002). Differently, the early sustained PIPR depends on the contributions of both outer and inner retinal photoreceptors (Adhikari et al., 2016; Hall and Chilcott, 2018). Considering that rods, cones, and mRGCs play different roles in mediating the PLR (McDougal and Gamlin, 2010; Rukmini et al., 2019), light stimuli can be designed to preferentially stimulate each photoreceptor class, thus providing a readout of their function (Kardon et al., 2009; Park and McAnany, 2015).

Melanopsin RGCs belong to the most recently identified cell type in the ganglion cell layer of the mammalian retina (Do and Yau, 2010; Hannibal et al., 2017) and represent the third photoreceptor of the eye. These intrinsically photosensitive photoreceptors modulate the non-image-forming functions of the eye, which are mainly the circadian photoentrainment and PLR, via projections to the hypothalamic suprachiasmatic and OPN, respectively (Hattar et al., 2002).

These cells are characterized by a unique property, which is the capability of firing without fatigue in response to continuous stimulation (La Morgia et al., 2018), consistent with their intrinsic activation (Berson et al., 2002; Hattar et al., 2002). In particular, the PIPR magnitude measured after 1.7 s (s) from offset of the light stimulus is considered a specific measure of mRGC function (Adhikari et al., 2015, 2016). In this context, chromatic pupillometry has been used to evaluate this cellular system in several clinical settings, including blinding disorders such as hereditary optic neuropathies (Kawasaki et al., 2010; Moura et al., 2013; Kawasaki et al., 2014; Munch et al., 2015; Nissen et al., 2015; Ba-Ali and Lund-Andersen, 2017; Loo et al., 2017), glaucoma (Kankipati et al., 2011; Rukmini et al., 2015; Kelbsch et al., 2016; Najjar et al., 2018, 2023) and neurodegenerative disorders such as Parkinson’s disease (PD) (Joyce et al., 2018; Feigl et al., 2020; Tabashum et al., 2021; Gaynes et al., 2022), idiopathic/isolated rapid eye movement sleep behavior disorder (iRBD) (La Morgia et al., 2022; Steiner et al., 2022), and Alzheimer’s disease (AD) (Oh et al., 2019; Romagnoli et al., 2020; La Morgia et al., 2023).

The purpose of this article is to briefly review the key steps of chromatic pupillometry protocols for studying *in-vivo* mRGC system functionality and provide the main findings of this technique in the research setting on neurodegeneration.

### 1.1 Search criteria

A PubMed literature search was conducted to identify the human studies available in the literature about chromatic pupillometry in Alzheimer’s disease. In order to provide a comparison with other neurodegenerative disorders, studies on chromatic pupillometry in Parkinson’s disease and isolated REM behavior disorder, were also included in the review. Conversely, works regarding achromatic pupillometry in these pathologies were excluded. The methodology used for the literature search consisted of a thorough search in PubMed including the following keywords, isolated and in combination: “melanopsin,” “pupillometry,” “chromatic pupillometry,” “pupil,” “Alzheimer,” “Parkinson,” and “REM behavior disorder.”

## 2 Key steps of a chromatic pupillometry protocol for *in-vivo* mRGC system evaluation

Chromatic pupillometry (also termed color pupillometry or selective wavelength pupillometry) refers to the evaluation of pupillary response to different wavelengths and intensities of light in order to differentiate outer and inner retinal photoreceptor-dependent contributions to the pupillary light reflex (PLR) (Rukmini et al., 2019). Different protocols, using different light paradigms and experimental settings of stimulation, have been tested and are now established to assess *in-vivo* PLR contribution mediated by melanopsin retinal ganglion cells (mRGCs) (Park et al., 2011; Adhikari et al., 2015; Chougule et al., 2019; Kelbsch et al., 2019; Rukmini et al., 2019).

Melanopsin-dependent pupillary responses are short-wavelength sensitive, have a higher threshold of activation, and are much slower and sustained compared with rod- and cone-mediated responses, dominating the tonic component of the PLR during exposure to high-irradiance and continuous light stimulus (Rukmini et al., 2019). Indeed, mRGCs contribute mainly to the tonic component of the post-illumination pupil response (PIPR) to bright blue light flash that persists after light stimulation is switched off (Adhikari et al., 2015). Given the role of mRGCs in circadian photoentrainment, the use of chromatic pupillometry to perform a functional evaluation of mRGcs may be proposed as an early biomarker of mRGC dysfunction in neurodegenerative disorders characterized by circadian and/or sleep dysfunction such as Alzheimer’s disease (AD), Parkinson’s disease (PD), and its prodromal stage, idiopathic/isolated rapid eye movement sleep behavior disorder (iRBD) (Wu and Swaab, 2007; Oosterman et al., 2009; Doppler et al., 2017; Ortuno-Lizaran et al., 2018). Specifically, chromatic pupillometry protocols aimed at detecting mRGC function can be categorized as those using short-duration light stimuli (e.g., light flashes or pulses) or those using continuously presented light stimuli (e.g., >30 s) (Rukmini et al., 2019).

**Figure 1:**
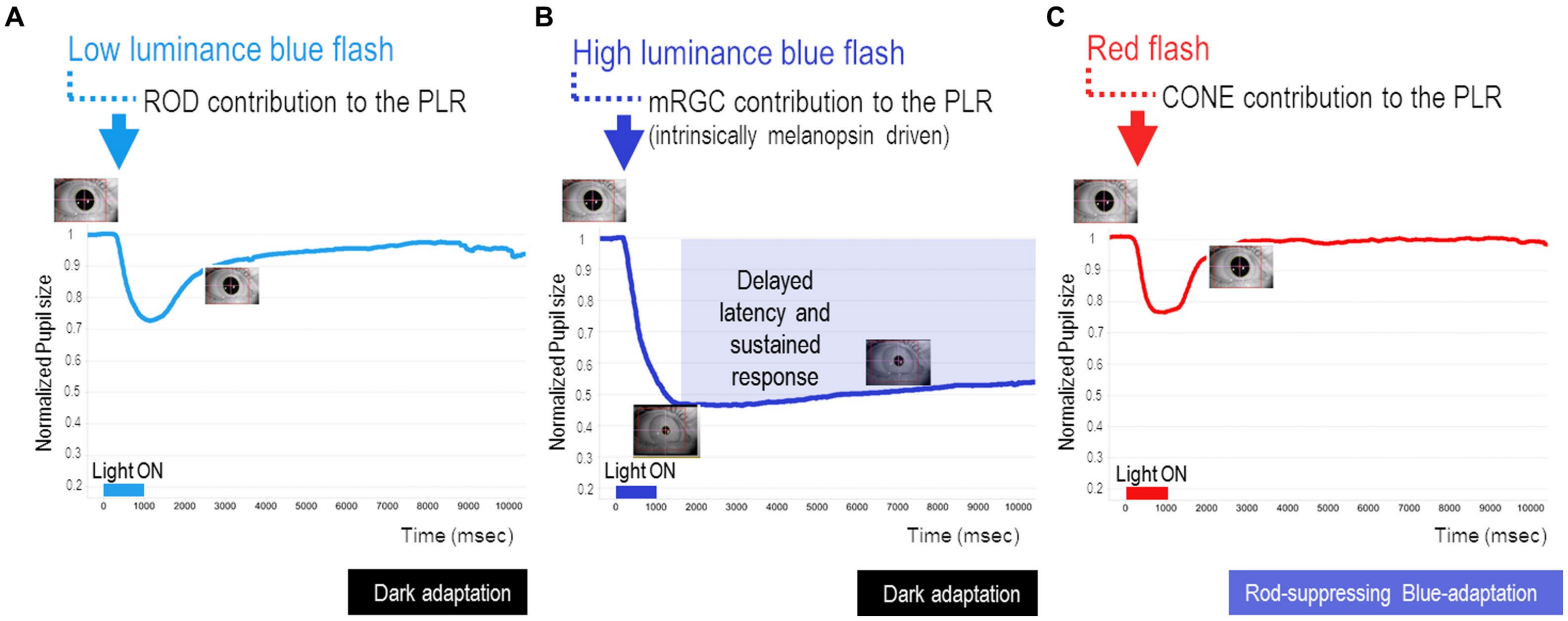
Key steps of chromatic pupillometry protocol for assessing retinal photoreceptor contributions to the human pupil light reflex (PLR). For our routine experimental research setting, we considered the following conditions, as previously reported (Park et al., 2011): **(A)** Rod-condition: low luminance (0.001 cd/m^2^) blue light flash presented in the dark. **(B)** Melanopsin-condition: photopically matched red and blue light flashes (450 cd/m^2^) presented in the dark. **(C)** Cone-condition: red light flash (10 cd/m^2^) presented on the rod-suppressing blue adapting field (6 cd/m^2^). Stimuli consisted of short wavelength (blue, dominant wavelength of 460–485 nm; mid = 472 nm) and long wavelength (red, dominant wavelength of 620–645 nm; mid = 632 nm) light flashes of 1 s (s) duration. The integrated pupillometer system measured the pupil diameter at a 100 Hz sampling frequency. The interstimulus interval (ISI) was 20 s for the rod- and cone-conditions (for both red and blue stimuli), while for the melanopsin condition, ISI was 30 s for the red stimulus and 70 s for the blue one. All recordings were completed in the same order with the red stimulus followed by the blue. For all three conditions, each stimulus was presented three times consecutively and the individual responses were obtained by their average recording.

One of these established standardized protocols takes about 30 min after a 10-min period of dark adaptation and implies a series of short-duration light (1 s) exposures (470 nm blue/640 nm red light) to assess the rod- (Figure 1A), melanopsin- (Figure 1B), and cone- (Figure 1C) PLR contribution (Park et al., 2011). In our clinic, we routinely adopted this protocol for research purposes in patients affected by different neurodegenerative disorders to ensure as much as possible compliance of these patients given its use of a short-duration stimulus (Figures 1A–C). Details of this protocol are fully described elsewhere (Park et al., 2011), but here we provide the main methodological steps:

1. Monocular recording of the dominant eye (contralateral one covered with a patch);
2. 10 min of dark adaption to naturally dilate the eye prior to chromatic pupillometry testing;
3. Colored light stimuli presentation by using a Ganzfeld ColorDome full-field stimulator (Espion V6, ColorDome Desktop Ganzfeld; Diagnosys LLC, Lowell, MA, United States) with an integrated pupillometer. In particular, mRGC contribution to the PLR was specifically assessed employing high-intensity photopically matched red and blue stimuli (450 cd/m^2^) presented in the dark. Specifically, stimuli consisted of long wavelength (red, dominant wavelength of 620–645 nm; mid = 632 nm) and short wavelength (blue, dominant wavelength of 460–485 nm; mid = 472 nm) light flashes of 1 s (s) duration. The integrated pupillometer system measured the pupil diameter at a 100 Hz sampling frequency. The interstimulus interval (ISI) was 30 s for the red stimulus and 70 s for the blue one. All recordings were completed in the same order with the red stimulus followed by the blue. Each stimulus was presented three times consecutively and the individual responses were obtained by their average recording.
4. Data quality control and analysis.

Since the eye-tracking systems may vary considerably in their sampling rate, precision, and noise susceptibility, as well as in the way they mark missing data, it is important to inspect the signals and efficacy of the preprocessing pipeline prior to analyzing the pupil size data. Indiscriminate inclusion of all available data or the use of non-robust outlier rejection methods may result in unnecessarily contaminated datasets, which could lead to incorrect interpretations of the collected data (Kret and Sjak-Shie, 2019). Briefly, the preprocessing pipeline (Kret and Sjak-Shie, 2019) can be broken down into:

(I) Preparing the raw eye-tracker output;
(II) Filtering the raw data: by doing so, we are able to identify three types of often occurring invalid pupil size samples (Kret and Sjak-Shie, 2019), namely, dilation speed outliers and edge artifacts, trend-line deviation outliers, and temporally isolated samples.
(III) Upsampling and smoothing the valid samples;
(IV) Splitting the data into the relevant segments and analyzing each segment individually.

**Figure 2:**
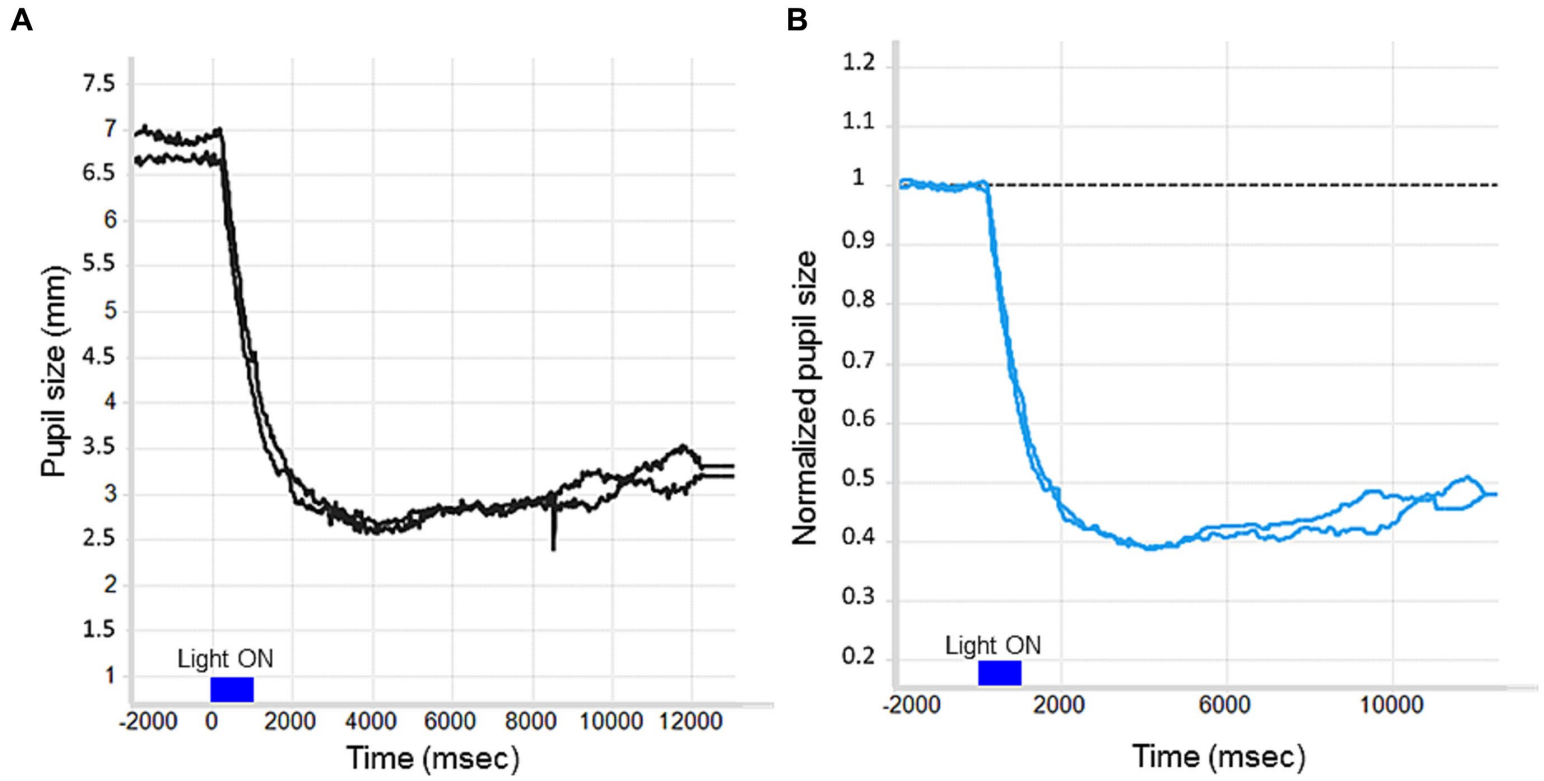
The effect of baseline correction on a raw PLR trace. **(A)** No baseline correction. **(B)** Divisive baseline correction: The Y-axis reflects proportional pupil size change relative to the baseline period.

In particular, our approach to pupil data preprocessing was to apply a median filter to remove the background noise and the noise given by the eye blink artifacts. Then, we proceeded with the baseline correction (i.e., analyzing changes in pupil size relative to a baseline period), which improves statistical power by taking into account random fluctuations in pupil size over time and controls for individual differences in pupil diameter (Mathot et al., 2018; Kelbsch et al., 2019) (Figures 2A,B). In this regard, we usually normalized the filtered pupil traces by the median pupil size during the 2 s in darkness preceding each light stimulus onset (Figure 2B). First, the median pupil size during the 2 s in darkness just before light exposure was taken as baseline pupil size, then all pupil sizes were divided by this baseline diameter and this was done separately for each trial (normalized pupil size = pupil size/baseline).

## 3 Chromatic pupillometry mRGC system metrics and their interpretation

Pupillary light reflex (PLR) can be used as an alternative and non-invasive tool to evaluate photoreceptor retinal functions (Maynard et al., 2015).

There are different metrics that could be used to quantify the contribution of rods and cones on human PLR as well as of the melanopsin retinal ganglion cells (mRGCs) (Adhikari et al., 2015). In particular, rod/cone metrics are categorized under the name of “PLR metrics” since they are calculated during the transient pupil constriction being elicited during the light stimulation under the corresponding protocol conditions, while the established marker of direct, intrinsic melanopsin activity is the post-illumination pupil response (PIPR), the sustained pupil-constriction after light offset (Dacey et al., 2005; Wong, 2012; Adhikari et al., 2015).

The PLR signal is quite robust, and metrics could be directly derived from raw data. However, the noise intrinsic to any electrophysiological recording suggests deriving PLR/PIPR metrics not only from raw data but also fitting the collected pupil data to a predefined model (linear, logarithmic, or exponential), and using the related parameters to extract the salient metrics.

**Figure 3:**
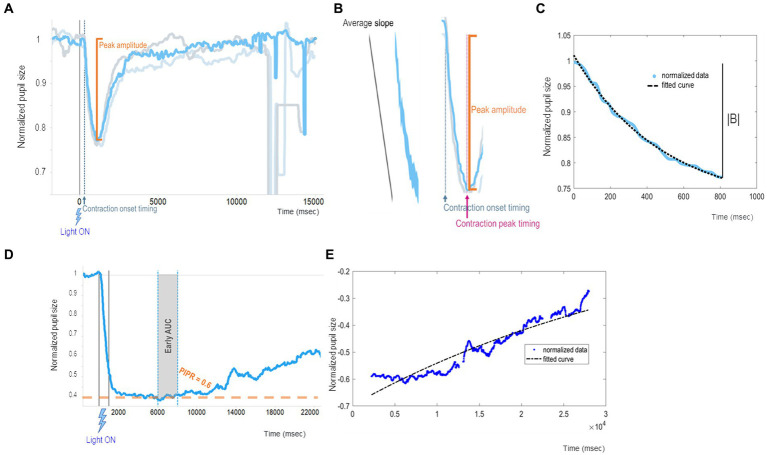
Example of chromatic pupillometry data analysis mRGC system metrics. **(A–C)** show examples of PLR metrics that mainly concern the onset of pupil contraction (which has a timing t1 and an amplitude y1 equal to the baseline) and its maximum constriction (which has a timing t2 and an amplitude y2, too) from which it is possible to calculate other metrics such as contraction onset timing (t1), contraction duration (t2-t1), peak amplitude [equal to the contraction amplitude (y2-y1)], and average slopes (y2-y1)/(t2-t1). In particular: **(A)** shows peak amplitude (or transient peak amplitude), which is defined as the difference between the normalized baseline and the minimum normalized PLR after light-stimulus onset (pupil maximum constriction); **(B)** shows an example of normalized PLR trace (azure line) and the contraction onset timing (latency relative to the stimulus delivery needed for the pupil to start constriction) and average slope (computed as the ratio between the peak amplitude and the duration of the contraction) parameters; **(C)** shows an example of a fitted PLR curve (black curve) by using the negative exponential model $y\;\left(t\right)=A-B\times e^{-\lambda \times t}$ where A is a constant, λ is the constriction velocity (global rate constant), and t is time in msec. **(D,E)** show examples of PIPR metrics evaluating mRGC sustained response entity and its dynamics: **(D)** shows an example of normalized PLR trace (azure line) with PIPR and early AUC (in gray shadow) parameters, where PIPR is calculated as the difference between the normalized baseline and the median normalized PLR measured over a 5–7 s time interval from the stimulus offset. **(E)** shows an example of an exponential modeled curve (black curve) during the mRGC-sustained response (redilation phase).

We summarize our recently published chromatic pupillometry metrics (La Morgia et al., 2023) as indirect and direct measures of mRGC contribution to the PLR (Table 1 and Figure 3). Generally speaking, the PLR entity and its dynamics could be measured by two approaches. One approach is to quantify the PLR starting from real data resulting from the chromatic pupillometry experiment by calculating parameters such as peak amplitude, contraction onset timing, average slope, and PIPR (Figures 3A,B,D). On the other hand, we may also adopt an approach based on best-fitting (the process of fitting experimental data to a specific mathematical model whose parameters are optimized) to obtain parameter estimates as the global rate constant of an exponential function (Figures 3C,E).

**Table 1:** Description and definition of the proposed PLR and PIPR metrics.

Metrics	Definition
PLR metrics	
Peak amplitude	Difference between the normalized baseline and the minimum normalized PLR after light-stimulus onset
Contraction onset timing	Time taken to start pupil constriction from the light-stimulus onset
Average slope	$\frac{\mathrm{Peak}\;\mathrm{amplitude}}{\mathrm{Contraction}\;\mathrm{peak}\;\mathrm{timing}-\mathrm{Contraction}\;\mathrm{onset}\;\mathrm{timing}}$
Constriction velocity	By negative exponential fitting of the constriction phase of the PLR curve in the form: $y\left(t\right)=A-B\times e^{-\lambda \times t}$ where A is a constant, λ is the constriction velocity (global rate constant), and t is time in msec
PIPR metric	
PIPR	Difference between the normalized baseline and the median normalized PLR measured over a 5–7 s time interval from the light-stimulus offset
Early AUC	Area under the curve over 5–7 s time interval from the light-stimulus offset
Redilation velocity	The global rate constant of the exponential fitted mRGC sustained response curve during the redilation phase

To describe in detail these chromatic pupillometry outcomes, peak amplitude (the maximum pupil constriction entity) was defined as the difference between the normalized baseline and the minimum normalized PLR around 700–1,500 milliseconds (msec) from the light-stimulus onset (Figure 3A), and the contraction onset timing was defined as the time taken to start pupil constriction from light-stimulus onset (Figure 3B). Considering these two PLR metrics, a larger PLR would be the one with a higher value of peak amplitude and a smaller value of contraction onset timing. In addition, we can calculate the average slope as the ratio between the aforementioned peak amplitude and time between the contraction peak timing and the contraction onset timing, as a metric indicative of the pupil constriction velocity (Figure 3B).

As an alternative to the univariate analysis, we can fit the contraction phase to a negative exponential model $y\;\left(t\right)=A-B\times e^{-\lambda \times t}$ , where A−B represents the initial value obtained when setting time (*t*) to 0. In turn, the asymptote reached at an infinite time is A, thus B represents the ideal extent of the contraction in case of an infinite stimulation. Then, λ is the time constant of the exponential curve, i.e., a parameter reflecting the speed of the pupil contraction. Figure 3 recapitulates all the abovementioned parameters with graphical examples.

Moreover, there are several metrics to quantify mRGC intrinsic sustained response, starting from real data as well as based on exponential best-fitting (Adhikari et al., 2015). Based on our previous publications (Romagnoli et al., 2020; La Morgia et al., 2023), the parameters for assessing mRGC sustained response produced by 1 s (s) bright blue light-flash could be the so-called PIPR, early area under curve (AUC) and redilation velocity (Figures 3D,E). PIPR parameter was defined as the difference between the normalized baseline and the median normalized PLR measured over a 5 to 7 s time interval from the light-stimulus offset. A specular metric to PIPR was the AUC estimated early in relation to the same time interval. By respecting these metrics, a larger mRGC sustained response corresponds to larger values of 5–7 s PIPR and early AUC. Mirroring the above case, the dynamics of intrinsic mRGC activity could be estimated via exponential fitting of the pupil redilation phase with the estimation of the exponential coefficient of the best-fitted curve. A larger mRGC sustained response would be defined by higher values of PIPR and early AUC, and by smaller values of redilation velocity as indexed by the time constant of the exponential curve.

## 4 The potential of chromatic pupillometry technique in the setting of AD research

Chromatic pupillometry has been proposed as a tool to specifically evaluate melanopsin retinal ganglion cell (mRGC) dysfunction in some neurodegenerative disorders, particularly in Alzheimer’s disease (AD), as in AD the presence of retinal ganglion cell (RGC) loss has been already documented (Hinton et al., 1986; Curcio and Drucker, 1993) as well as the occurrence of abnormal circadian photoentrainment (La Morgia et al., 2017).

A great deal of studies are available on animal models and these are characterized by circadian and sleep dysfunction even in the early phases of the disease (Uddin et al., 2020). Based on this, an early mRGC dysfunction or loss, which has been already demonstrated in *post-mortem* AD retinas in humans (La Morgia et al., 2016), may be envisaged as a contributor to the circadian and sleep dysregulation in these patients (La Morgia et al., 2017).

AD is the most frequent cause of dementia, characterized by abnormal accumulation of misfolded amyloid-ß (Aß) protein and hyperphosphorylated tau in the brain. Cognitive impairment and memory loss are the most prominent features of the disease, nevertheless, visual and sleep disturbances are frequently reported from the disease’s early phases (Uddin et al., 2020). Besides the pathological involvement of the visual cortex, histological hallmarks of AD in the form of amyloid extracellular plaques have also been demonstrated in the eye, especially in the inner retina, in *post-mortem* tissues from patients with AD (Koronyo et al., 2017). The resulting degeneration of RGCs with macular and optic nerve thinning was also confirmed by several *in-vivo* studies with optic coherence tomography (OCT) (Chen et al., 2023). These studies demonstrated a retinal nerve fiber layer (RNFL) thinning mostly in the superior and inferior sectors of the optic nerve, suggesting a preferential loss of the magnocellular component of the optic nerve in AD (Chan et al., 2019). Interestingly, the presence of amyloid pathology has also been demonstrated within the mRGCs in *post-mortem* AD human retinas, as well as the presence of morphological alterations in the surviving mRGCs, such as dendrite varicosities and reduced arborization (La Morgia et al., 2016). This suggests that an early mRGC dysfunction or loss may contribute to the circadian and sleep dysregulation frequently observed in these patients.

The current recommendations for AD diagnosis include tau/amyloid cerebrospinal fluid (CSF) and positron emission tomography (PET) imaging (Dubois et al., 2021), but there is a still unmet need for easily accessible and objective biomarkers either for biological definition of AD and its prodromal phase, the amnestic mild cognitive impairment (aMCI), as well as for stratifying the risk of conversion from aMCI to AD. To improve diagnostic capabilities for AD and aMCI, the diagnostic workup should include laboratory- and/or instrumental-measured early biomarkers; however, the routine use of biomarkers in the clinical setting is not yet recommended for both conceptual and evidence-based reasons (Dubois et al., 2021). In this framework, considering the role of mRGCs in circadian photoentrainment, the use of chromatic pupillometry to perform a functional evaluation of mRGcs may be a potential biomarker of mRGC dysfunction and, thus, of circadian dysfunction in both AD and aMCI.

Besides chromatic pupillometry, non-chromatic pupillometry was variably used in patients with AD, not specifically addressing the mRCG contribution to the pupillary light reflex (PLR) but mainly the cholinergic/parasympathetic dysfunction typical of AD. The results obtained have highlighted significant changes in terms of latency, amplitude, maximum constriction velocity, and acceleration in patients with AD, but did not find any significant difference in PLR in preclinical AD compared with controls (Chougule et al., 2019). Interestingly, Granholm and others studied pupillary diameter during a cognitive task, as a psycho-physiological biomarker of early risk for mild cognitive impairment (MCI) or AD, suggesting that pupillary changes may underlie subtle cognitive abnormalities that can help differentiate between MCI subtypes and identify subjects at risk of AD (Granholm et al., 2017).

mRGC investigation in humans, in particular at early disease stage (Hannibal et al., 2004; La Morgia et al., 2010; Esquiva et al., 2017; Hannibal et al., 2017; Ortuno-Lizaran et al., 2018; Esquiva and Hannibal, 2019; La Morgia et al., 2023), remains challenging due to objective difficulties of *in-vivo* mRGC system exploration and high variability in terms of technical protocols. A few studies that utilized chromatic pupillometry have been carried out on both AD and pre-symptomatic patients in recent years (Table 2). Oh et al. focused on the contribution of mRGC to PLR using intense (2.3 log cd/m^2^) red (620 nm) and blue (450 nm) light stimuli. PLR assessment was coupled with actigraphic recordings of the sleep–wake cycle in a group of 10 pre-symptomatic AD patients, defined as cognitively healthy but with abnormal Aβ42/tau CSF ratio compared to 10 healthy controls (Oh et al., 2019). Comparative analysis failed to disclose any significant difference in both pupillometric and actigraphic results in patients compared to controls. Nevertheless, higher variability of sustained pupillary response to blue light and in the circadian rhythm was observed in pre-symptomatic AD patients compared to controls, suggesting that a change in mRGC function may be present even before clinical symptoms become evident.

**Table 2:** Summary of studies on chromatic pupillometry in Alzheimer’s disease and in synucleinopathies.

Study reference	Clinical focus	Light paradigm	Main findings
Oh et al. (2019)	Pre-symptomatic AD (*n* = 10) Controls (*n* = 10)	Red (620 nm) and blue (450 nm) light stimuli Intensity 2.3 log cd/m^2^ Duration 1 s	No significant differences between groups, higher variability of sustained PLR to blue light in pre-symptomatic AD compared to control
Romagnoli et al. (2020)	Mild–moderate AD (*n* = 26) Controls (*n* = 26)	Red (632 nm) and blue (472 nm) light stimuli: -Rod-condition: blue light, low intensity 0.001 cd/m^2^-Melanopsin-condition: red and blue light flashes, high intensity 450 cd/m^2^ Cone-condition: red light (10 cd/m^2^) against blue adapting field (6 cd/m^2^) Duration 1 s	Significant difference in rod-mediated transient peak amplitude in the AD group compared to controls Higher variability of PIPR in the AD group, despite not being statistically significant
La Morgia et al. (2023)	Mild–moderate AD (*n* = 29) Controls (*n* = 26)	Same as Romagnoli et al., 2020	AD patients (rod-condition): significantly delayed onset of transient PLR response, lower average slope, and also a significantly reduced |B| indicative of a lower exponential decay
Kawasaki et al. (2020)	Early AD (*n* = 16) Controls (*n* = 16)	2 blue (470 nm) light stimuli, intensity 1.75 and 2.23 log cd/m^2^ (0 log = 1cd/m^2^) 5 red (633 nm) light stimuli, intensity 0, 0.5, 1, 1.5 and 2.6 log cd/m^2^ (0 log = 1cd/m^2^) Duration 1 s	Significantly smaller baseline pupil size in the AD group and no significant difference in pupillary response to all red/blue lights
Joyce et al. (2018)	PD (*n* = 17) Controls (*n* = 12)	Blue (465 nm) and red (638 nm) light stimuli Irradiance 15.1 log photons.cm^−2^.s^−1^ Duration: pulsed (8 s rectangular) or phasic (12 s, 0.5 Hz sinusoidal)	Reduced PIPR amplitude for both pulsed and phasic blue stimulation in patients with PD compared to controls
Feigl et al. (2020)	PD (*n* = 30) Controls (*n* = 29)	Blue (460 nm), green (519 nm) and red (630 nm) light stimuli Irradiance 15.5 log.quanta.cm^−2^.s^−1^	Reduced PIPR to blue and green stimuli in patients with PD compared to controls Correlation between lower PIPR amplitudes with poor sleep quality and decreased RNFL thickness
Tabashum et al. (2021)	PD (*n* = 19) Controls (*n* = 10)	Blue (470 nm) and red (610 nm) light stimuli High energy 30 μW and low energy 8 μW Duration 5 s	Significant difference in net PIPR and net PIPR% in patients with PD compared to controls
Gaynes et al. (2022)	PD (*n* = 17) Controls (*n* = 9) Parkinsonism (*n* = 2)	Blue (470 nm) and red (640 nm) light stimuli Irradiance 8 and 30 μW·cm^−2^·nm^−1^ Duration 5 s	Altered PIPR with blue high irradiance stimuli in patients with PD Abnormal pupil latency at both blue and red stimuli in patients with PD
Steiner et al. (2022)	iRBD (*n* = 69)	Blue light pulses (465 nm) Intensity 56 cd/m^2^ Duration 1 s	Significantly reduced PIPR in iRBD patients with mild neurocognitive disorder compared with patients with iRBD only Significant correlation of PIPR with cognitive performance, more pronounced in patients with lower dopamine-transporter density
La Morgia et al. (2022)	iRBD (*n* = 16) Controls (*n* = 16)	Same as Romagnoli et al., 2020	Higher baseline pupil diameter and decreased rod-mediated peak amplitude in patients with iRBD compared to controls Decreased rod-mediated peak amplitude in patients with iRBD with evidence of p-α-syn deposition at skin biopsy No difference in mRGC-mediated PIPR between groups Correlation of the rod-mediated peak amplitude with REM atonia index

AD, Alzheimer’s disease; PD, Parkinson’s disease; iRBD, isolated REM sleep Behavior Disorder; PLR, pupillary light response; PIPR, post-illumination pupil response; RNFL, retinal nerve fiber layer; mRGCs, melanopsin retinal ganglion cells; p-α-syn, phosphorylated-α-synuclein.

Our group applied the above-described chromatic pupillometry protocol to assess separately the contribution of rods, cones, and mRGCs to the pupillary response in 26 AD patients in the mild–moderate stage compared to 26 controls (Romagnoli et al., 2020). A higher variability of post-illumination pupil response (PIPR) was observed in the AD group, despite not being significantly different from controls. Conversely, a significant difference in rod-mediated transient peak amplitude was observed in AD, suggesting that in early stages, the AD pathology may affect primarily the mRGC dendrites before involving the cell body, thus confirming the aforementioned histological findings (La Morgia et al., 2016).

In order to perform a multimodal investigation of the mRGC system, those patients were further evaluated with OCT, actigraphy recording of the sleep–wake cycle, and brain functional MRI (fMRI) with visual and cognitive stimulation, and compared to controls (La Morgia et al., 2023). The fMRI visual stimulation consisted of periods of illumination with blue or red light (50 s) alternated with darkness (20 to 30 s). The visual paradigm was also combined with cognitive stimulation during fMRI (consisting of an auditory task) to evaluate sustained attention and the effects of the interaction between light stimulation and cognitive task. The OCT analysis failed to show significant differences between AD and controls except for a thinner inferotemporal sector at the ganglion cell layer level corresponding to the superonasal sector of the optic nerve in AD compared to controls. Overall, pupillometry results confirmed the lack of significant differences under the mRGC condition, despite a significantly delayed onset with a lower average slope and reduced amplitude of the transient PLR under rod-condition in the AD group. Actigraphy recording, even if not significantly different, showed higher variability for circadian measures in patients with AD compared to controls. Under the fMRI visual paradigm, patients with AD showed a reduced occipital cortex activation to blue light compared to red light, while, under the visual-cognitive paradigm, the same patients showed a tendency toward an improvement of the cognitive performances under blue light stimulation, even if not statistically significant. Overall, these multimodal findings confirm once again that mRGC dysfunction is central in AD since its early stages, but the progression of this process, from dendropathy to cell death, may be variable among patients (La Morgia et al., 2023).

Finally, Kawasaki et al. performed a pilot sub-study from a prospective study on biomarkers in the early stages of AD, using chromatic pupillometry under photopic conditions to assess primarily cones and melanopsin-mediated pupillary responses. In the study, 16 early AD subjects were compared with 16 controls, showing significantly smaller baseline pupil size, while pupillary responses were not significantly altered, nor correlated with MMSE score, MRI hippocampal volume, or CSF biomarkers. Nevertheless, a trend between absolute hippocampal volume and the blue light PIPR was noted, suggesting that, despite mRCG dysfunction not being detectable with pupillometry in early stages, the PIPR may be an indirect marker of hippocampal atrophy in AD (Kawasaki et al., 2020).

## 5 Chromatic pupillometry findings in synucleinopathies

Furthermore, chromatic pupillometry to assess melanopsin retinal ganglion cell (mRGC) function has also been applied to other neurodegenerative disorders, such as Parkinson’s disease (PD) and its prodromal stage, idiopathic/isolated rapid eye movement sleep behavior disorder (iRBD) (Joyce et al., 2018; Feigl et al., 2020; Tabashum et al., 2021; Gaynes et al., 2022; La Morgia et al., 2022; Steiner et al., 2022).

PD is a complex neurodegenerative disease caused by α-synuclein deposition in the brain that aggregates in the form of Lewy bodies within the neurons and leads to cellular death. Non-motor symptoms, including olfactory deficit, sleep and circadian disturbances, depressed mood, and cognitive impairments are also frequent and may precede motor symptoms. The etiology underlying sleep and circadian disturbances in PD is not well understood; one hypothesis includes dysregulation of the circadian rhythms due to reduced dopaminergic neurotransmission. Moreover, since mRGCs project to brain areas involved in arousal and sleep regulation, their dysfunction may underpin the circadian and sleep disturbances observed in PD (La Morgia et al., 2017). Interestingly, mRGCs are linked to dopaminergic amacrine cells both pre- and post-synaptically (Hannibal et al., 2017). Dopamine has a role in the light-adaptation process, upregulating melanopsin transcription in mRGCs and increasing their photosensitivity (Sakamoto et al., 2005). Thus, the already demonstrated loss of dopaminergic amacrine cells in PD (Ortuno-Lizaran et al., 2020) is expected to cause a reduction of melanopsin expression with dysfunction or loss of mRGCs, consequently altering their contribution to pupillary light reflex (PLR). We here briefly review the latest works on chromatic pupillometry in PD (Table 2).

Joyce et al. evaluated melanopsin and rod/cone contributions to the pupil response in 17 patients with PD and 12 controls using a chromatic pupillometry protocol with pulsed or phasic short (blue) and long (red) wavelength light stimuli. Pupillary unrest in darkness was also used as a measure of autonomic tone. Patients were furthermore assessed for disease severity (UPDRS, H&Y), cognitive impairment (MMSE), sleep quality (Pittsburgh Sleep Quality Index questionnaire), and peripapillary retinal nerve fiber (RNFL) thickness. Patients with PD showed reduced post-illumination pupil response (PIPR) amplitude for both pulsed and phasic blue stimulation compared to controls, while both groups presented similar pupillary unrest. PIPR amplitudes did not correlate with disease severity, sleep quality, or RNFL thickness. These results suggested that melanopsin’ contribution to the pupil response is impaired in early-stage PD without clinically evident ophthalmic abnormalities (Joyce et al., 2018).

Feigl et al. assessed PLR and PIPR using chromatic pupillometry in 30 patients with PD and 29 healthy controls. Subjects also underwent ophthalmic examination including optic coherence tomography (OCT) and assessment of circadian rhythm using actigraphy, dim light melatonin onset, and sleep questionnaires. Patients with PD showed significantly reduced melanopsin-mediated PIPR amplitudes, correlating with poor sleep quality, RNFL thinning, and earlier melatonin onset. This suggests that reduced and irregular inputs to the suprachiasmatic nucleus via dysfunctional mRGCs are responsible for poorer sleep in patients with PD (Feigl et al., 2020).

Tabashum et al. measured pupillary diameter variation of the contralateral eye to red and blue light stimuli using automated tracking with a Kalman filter. In all, 19 PD and 10 control subjects were tested. PIPR (pre-stimulus pupil diameter – post-stimulus pupil diameter) and net PIPR (blue PIPR – red PIPR) were calculated, along with two other measures normalized by the pupil diameter: PIPR% (PIPR*100/pre-stimulus pupil diameter) and net PIPR% (blue PIPR% – red PIPR%). Statistical analysis showed a significant difference in net PIPR and net PIPR% in patients with PD compared to controls, suggesting that net PIPR can be used as a potential biomarker for PD (Tabashum et al., 2021).

More recently, Gaynes et al. recorded consensual PIPR in the left eye after 5-s pulses of blue and red light stimuli to the right dilated eye. In all, 17 PD subjects, 9 controls, and 2 subjects with parkinsonism presumed to have Lewy body dementia and multiple system atrophy were compared. Subjects with PD variably demonstrated altered PIPR with short-wavelength high irradiance stimuli, consistent with mRGC dysfunction, and abnormal pupil latency at both short- and long-wavelength stimuli, while subjects with parkinsonism did not show any pupillary changes (Gaynes et al., 2022).

iRBD represents the strongest prodromal risk factor for α-synucleinopathies, characterized by loss of muscle atonia during REM sleep, leading to abnormal sleep behaviors ranging from simple muscular twitches to complex, sometimes even violent, limb and body movements. Reduced pupil constriction and dilation have been demonstrated both in patients with RBD and PD in comparison to controls (Perkins et al., 2021), while chromatic pupillometry has been used by only two studies in iRBD (La Morgia et al., 2022; Steiner et al., 2022). Steiner et al. (2022) evaluated the melanopsin-mediated PIPR with cognition (CERAD-plus) in 69 patients with iRBD. PIPR was significantly correlated with cognitive functions, especially executive functioning, and this was more evident in patients with lower dopamine-transporter density. Patients with iRBD with mild neurocognitive disorder showed significantly reduced PIPR compared to those without. PIPR was then proposed as a potential biomarker for cognitive function in iRBD.

In our study, 16 patients with iRBD and 16 controls were tested to compare rod- and cone-mediated PLR and mRGC-contribution (PIPR). Pupillometric results were also correlated with clinical signs, REM atonia index (RAI), DaTscan, and skin deposition of phosphorylated-α-synuclein (La Morgia et al., 2022). Patients with iRBD presented higher baseline pupil diameter and decreased rod-mediated peak amplitude, while mRGC-mediated PIPR did not differ from controls. Interestingly, only patients with iRBD with evidence of phosphorylated-α-synuclein skin deposition presented a reduced peak amplitude (rod-condition) compared to controls. Moreover, the rod-mediated PLR correlated with RAI. These results suggest that PLR rod contribution is impaired in iRBD, probably reflecting early mRGC dendropathy, and that it can be considered as a potential biomarker for the risk of phenoconversion of the disease.

## 6 Final considerations on chromatic pupillometry in AD and other neurodegenerative disease settings

Chromatic pupillometry studies in the setting of neurodegenerative disorders have been limited to small sample sizes, the cross-sectional nature of the studies’ design, and methodological differences (Chougule et al., 2019). Larger longitudinal studies correlating pupillometric measures to circadian function in the prodromal stages of the diseases are needed to highlight the role of this cellular system in circadian dysfunction and prediction of disease severity (Mitolo et al., 2018; La Morgia et al., 2023).

In this context, a properly designed light stimulus is *a conditio sine qua non* for a good-quality study, since only an appropriate chromatic stimulus allows the quantification of the mRGC system functional deficit. Unfortunately, the scientific community active in this research field has not yet reached a consensus on a standardized chromatic pupillometric methodology for the evaluation of mRGC-mediated pupil function, both in terms of experimental setting and data manipulation/analysis pipeline, and this makes the results difficult to compare. Indeed, factors such as normalization of the pupillary response to the baseline pupil diameter, smoothing/filtering modes, or different algorithms used to calculate chromatic pupillometric endpoints may, independently or in combination, influence the magnitude/variability of the results (Mathot et al., 2018; Chougule et al., 2019; Kret and Sjak-Shie, 2019).

Although longitudinal studies are fundamental to show the impact of mRGC system dysfunction as the disease progresses, we suggest that research can continue to benefit also from cross-sectional studies, which would be less affected by their intrinsic limitations if based on age- and disease severity-matched comparison groups (Chougule et al., 2019).

Moreover, the evaluation by chromatic pupillometry of mRGC system functionality may lay the groundwork for a new, easily accessible biomarker that can also be exploited as the starting point for future longitudinal cohort studies aimed at stratifying the risk of conversion of these disorders.

We finally highlight that a large-scale multicentric validation of a chromatic pupillometry protocol is warranted, as this approach may have potential use in clinical trials aimed at correcting circadian/sleep disorders as a secondary preventive action of AD, providing an additional low-cost quantitative mRGC outcome, incorporated into easily testing systems.

## Author contributions

MR: Conceptualization, Data curation, Formal analysis, Investigation, Methodology, Resources, Software, Validation, Visualization, Writing – original draft, Writing – review & editing. GA: Data curation, Investigation, Methodology, Writing – original draft, Writing – review & editing. PA: Conceptualization, Data curation, Formal analysis, Investigation, Methodology, Writing – review & editing, Visualization. VC: Conceptualization, Investigation, Methodology, Supervision, Writing – review & editing, Funding acquisition. CLM: Conceptualization, Data curation, Formal analysis, Funding acquisition, Investigation, Methodology, Project administration, Resources, Software, Supervision, Validation, Visualization, Writing – review & editing.
